# Co-occurrence Interaction Networks of Extremophile Species Living in a Copper Mining Tailing

**DOI:** 10.3389/fmicb.2021.791127

**Published:** 2022-01-05

**Authors:** Gabriel Galvez, Jaime Ortega, Fernanda Fredericksen, Victor Aliaga-Tobar, Valentina Parra, Angélica Reyes-Jara, Lorena Pizarro, Mauricio Latorre

**Affiliations:** ^1^Laboratorio de Bioingeniería, Instituto de Ciencias de la Ingeniería, Universidad de O’Higgins, Rancagua, Chile; ^2^Departamento de Bioquímica y Biología Molecular and Advanced Center for Chronic Diseases (ACCDiS), Facultad de Ciencias Químicas y Farmacéuticas, Universidad de Chile, Santiago, Chile; ^3^Laboratorio de Microbiología y Probióticos, INTA, Universidad de Chile, Santiago, Chile; ^4^Laboratorio de Inmunidad Vegetal, Instituto de Ciencias Agroalimentarias, Animales y Ambientales, Universidad de O’Higgins, Rancagua, Chile; ^5^Laboratorio de Bioinformática y Expresión Génica, INTA, Universidad de Chile, Santiago, Chile

**Keywords:** extremophiles, microbiomes, co-occurrence networks, copper mining tailing, bacteria

## Abstract

Copper mining tailings are characterized by high concentrations of heavy metals and an acidic pH, conditions that require an extreme adaptation for any organism. Currently, several bacterial species have been isolated and characterized from mining environments; however, very little is known about the structure of microbial communities and how their members interact with each other under the extreme conditions where they live. This work generates a co-occurrence network, representing the bacterial soil community from the Cauquenes copper tailing, which is the largest copper waste deposit worldwide. A representative sampling of six zones from the Cauquenes tailing was carried out to determine pH, heavy metal concentration, total DNA extraction, and subsequent assignment of Operational Taxonomic Units (OTUs). According to the elemental concentrations and pH, the six zones could be grouped into two sectors: (1) the “new tailing,” characterized by neutral pH and low concentration of elements, and (2) the “old tailing,” having extremely low pH (~3.5) and a high concentration of heavy metals (mainly copper). Even though the abundance and diversity of species were low in both sectors, the *Pseudomonadaceae* and *Flavobacteriaceae* families were over-represented. Additionally, the OTU identifications allowed us to identify a series of bacterial species with diverse biotechnological potentials, such as copper bioleaching and drought stress alleviation in plants. Using the OTU information as a template, we generated co-occurrence networks for the old and new tailings. The resulting models revealed a rearrangement between the interactions of members living in the old and new tailings, and highlighted conserved bacterial drivers as key nodes, with positive interactions in the network of the old tailings, compared to the new tailings. These results provide insights into the structure of the soil bacterial communities growing under extreme environmental conditions in mines.

## Introduction

Most of the well-known bacteria are adapted to moderate physiological conditions, such as template temperatures, low metal concentrations, and mostly neutral pH. Over the years, our understanding of extremophile microorganisms adaptation to live and thrive under extreme conditions has been expanded, while microorganisms adapted to different extreme conditions millions of years ago (Antranikian et al., 2005). In this context, extremophiles can serve as a vestige to decipher how communities of species have adapted to environmental changes suffered throughout the Earth’s history, being a unique natural reservoir to understand the structure of interactions within a community which has survived extreme conditions.

Although there have been significant advances in the study of extremophiles, it still remains a rather complex topic of investigation due to difficulties in replicating the extreme conditions in which these organisms live (Orellana et al., 2018). In particular, mining environments, which exhibit a very acidic pH and high metal concentrations, have been widely studied over the years (Kishimoto et al., 1991; Bond et al., 2000).

In order to survive in the extreme miner environment, microorganisms inhabiting mining environments employ different mechanisms, such as changing the permeability of their membranes to delay proton entry to their cytoplasm and thus, control pH (Navarro et al., 2013). For heavy metal tolerance, bacteria can form biofilms to solubilize minerals or expel heavy metals from their cytoplasm (Alvarez and Jerez, 2004; Valdés et al., 2008). These mechanisms have been mainly studied in *Acidithiobacillus ferrooxidans*, a bacteria commonly found in mining environments (Akinci and Guven, 2011). Examples of other extremophiles that have been isolated from this environment are as: *Leptospirillum ferrooxidans*, *Acidiphilum* sp., *Acidithiobacillus ferrooxidans*, *Acidocella* sp., and members of the Proteobacteria phylum, among others (Edwards et al., 1999; He et al., 2007; Xie et al., 2007; González-Toril et al., 2011; Santofimia et al., 2013). Additionally, these extremophiles have been widely used in biomining throughout the years.

As currently known, a very interesting characteristic of these extreme microorganisms is that they form communities, in which each species complements each other to adapt to the environmental conditions. Specifically in the miner environment, some studies have described communities of a microbial community of a tungsten mine tailing (Chung et al., 2019), and the shift of the microbial community in a revegetated copper mine tailing (Yang et al., 2017), among others. In the same line, our group has described how an acidophilic bacterial community has adapted to its extreme environment and its bioleaching potential (Bordron et al., 2016; Latorre et al., 2016; Hart et al., 2018). These kinds of studies have also been made in acid mine drainages (Baker and Banfield, 2003; Teng et al., 2017; Zhang et al., 2021).

In general, the microbial community found in mines displays a low microbial abundance, with few microbial families or phyla covering the majority of the diversity (Tyson et al., 2004; Latorre et al., 2016). In this environment, most of the microorganisms that carry out the mineral degradation process are autotrophic; however, heterotrophic microorganisms are also present and live off the waste produced by the autotrophs (Rawlings, 2005). In addition, other microbial processes that allow an improved attachment to minerals have been studied, such as the degradation of organic metabolites by *Acidiphilium multivorum* to protect chemolithoautotrophs from the toxicity of these metabolites, or biofilm formation by *Leptospirillum*, *Acidithiobacillus*, and *Sulfobacillus* species among others (Latorre et al., 2016).

Among the set repertoire of extreme environments are those generated by humans, such as waste generated by mining activities. These wastes are mainly composed of finely ground rock and water and are known as mine tailings. These tailings often contain between 12 and 20% of the minerals that could not be recovered from the ore, and thus, contain a high concentration of heavy metals and an extremely low pH (Oluwasola et al., 2014). These characteristics make tailings favorable for finding unique acidophile and heavy metal-resistant extremophiles with potential biotechnological applications, which continues to be a poorly explored extreme scenario in terms of the characterization of their bacterial communities.

The study of bacterial communities usually begins with 16 s rRNA gene sequencing. With this data, Operational Taxonomic Units (OTUs) can be identified and co-occurrence networks can be constructed (Faust and Raes, 2016). The analysis of these networks can then be used to describe the different interactions between the members of the bacterial community, and the comparison between networks from different environments allows the identification of relevant species. Bacterial “drivers” are species that are poorly conserved among the different environments, while bacterial “stabilizers” correspond to those showing high conservation rates in different sites (Kuntal et al., 2019). This information can be useful to describe possible interdependencies between each member of bacterial communities.

In this study, we analyzed samples from six different zones belonging to the Cauquenes tailing, the largest copper waste deposit worldwide generated from “El Teniente” mine in the O’Higgins region in central Chile. In each sample, the heavy metal concentration and pH were determined. Next, total DNA extraction was carried out and sequenced, and OTUs were assigned to evaluate the different interactions of the bacterial communities in this tailing, through the construction of co-occurrence networks.

## Materials and Methods

### Sample Collection

Tree replicated soil samples (~100 g each) were collected in January 2020 (austral summer) under sterile conditions from the upper 5 to 10 cm of the soil surface at six distinctive zones: zones 1–6 (18 samples in total). Each replicate of soil was homogenized *in situ* before storage. From each replicate, a subsample of 50 g was immediately stored on dry ice for microbial analyses (stored during approximately 1 week), while another subsample of 50 g was air-dried, sieved (≤ 2 mm), and stored for physicochemical analyses.

### Soil DNA Extraction

DNA was extracted from subsamples collected from each of the three soil samples at each site using the Qiagen kit DNeasy Blood and Tissue, combining the manufacturer’s instructions and the CTAB-based method (Zhou et al., 1996). Five grams of soil were resuspended in 5 ml extraction buffer [100 mm Tris–HCl; pH 8, 100 mm Na EDTA; pH 8, 100 mm Na_2_HPO_4_, 1.5 M NaCl, and 1% (w/v) CTAB] and then 25 μl of proteinase K was added and mixed by vortex, followed by incubation at 65°C for 2 h, with constant mixing. The mixture was centrifuged at 4000 × g for 10 min at room temperature and the supernatant fluid was transferred to a clean tube, to which 0.5 volume of ethanol 100% was added. Then, the samples were vortexed for 10 s and the mixture was transferred into a DNeasy mini spin column to continue the kit protocol. Concentrations of DNA were evaluated with a Nanodrop. Microbial DNA was amplified using a bacteria-specific primer set, 28 F (5′-GA GTT TGA TCM TGG CTC AG-3′) and 519 R (5′-GWA TTA CCG CGG CKG CTG-3′), flanking variable regions V1-V3 of the 16 S rRNA gene (Turner et al., 1999), with barcode on the forward primer. Amplification was performed using the promega GoTaq^®^ G2 Flexi DNA Polymerase Mix, under the following conditions: initial denaturation at 94°C for 3 min, followed by 28 cycles, each set at 94°C for 30 s, 53°C for 40 s and 72°C for 1 min, with a final elongation step at 72°C for 5 min. After amplification, PCR products were checked in 2% agarose gels to determine the success of the amplification and then stored at −20°C until DNA analyses.

### Soil Physicochemical Measurements

For the measurement of nutrients and pH, five grams of sample were placed in a sterile 15 ml tube; then, 1:1 (w/v) distilled water was added (Thomas and Wimpenny, 1996). The samples were homogenized for 2 h at room temperature and then centrifuged at 12000 g for 5 min. The soluble fraction was recovered. The pH quantification was performed directly from the soluble fraction. Nutrient measurement was performed using 500 ul of the soluble fraction using the Total Reflection X-Ray Fluorescence spectroscopic technique, following a previously described protocol (Tapia et al., 2003).

### 16S rRNA Gene Amplification and Sequencing

Each sample was sent to the Mr. DNA company for amplification and subsequent sequencing of the 16S rRNA gene, using the Illumina^®^ MiSeq kit. This service was performed by sequencing the V3 and V4 hypervariability region. The sequences were processed following previously described protocols (Handl et al., 2011). The sequences were overlapped and grouped by samples, to later eliminate the “barcode” segments of the sequences. Sequences less than 150 bp or with ambiguous base allocation were not considered for further analysis. The sequences accepted as valid were grouped using the UClust algorithm (v2.22) with 4%, in order to eliminate chimeras and groups with a single sequence (singletons; Edgar, 2010). After being processed, the sequences obtained were analyzed with the Quantitative Insights Into Microbial Ecology 2 Program (Caporaso et al., 2011), using the “qiime feature-classifier classify-sklearn” protocol. The sequences were then compared against Greengenes databases (Mcdonald et al., 2011), using 97% similarity, directly assigning the taxonomy from the closest match. To assign the OTUs, they were filtered by a minimum of four reads (readings) per sample, eliminating those OTUs corresponding to mitochondria, chloroplasts, and not classified within the of bacterial range. The rarefaction analysis was performed using the “qiime diversity alpha-rarefaction” code, setting the number of readings at the lowest obtained from the samples. To obtain the alpha diversity, the Shannon and evenness index were calculated, using the code “qiime diversity core metrics phylogenetic.” To construct the phylogenetic tree, we generated a tree file from the previously taxa obtained using the “qiime phylogeny align-to-tree-mafft-fasttree” code and visualized the associations using the Interactive tree of life (ITOL) platform (Letunic and Bork, 2021). All sequence data used in this study have been deposited in the Sequence Read Archive of the National Center for Biotechnology Information under the BioProject accession number PRJNA769703.

#### Construction of Co-occurrence Networks

The construction and subsequent visualization of the networks were carried out with the Cytoscape (v3.7.1) program (Shannon et al., 2003), using the CoNet plugin (Faust and Raes, 2016). The networks were constructed with the abundance tables obtained from the processing and analysis of bacterial sequences. To examine the structural association between the OTUs and soil physicochemical and nutritional parameters, we included the pH, micro and macronutrients as network nodes. We selected these physicochemical and nutritional parameters for the network display because they unveiled the association of OTUs in the six sampled sites of tailings, revealing important factors for bacterial growth (Russell and Cook, 1995; Moore and Helmann, 2005; Porcheron et al., 2013). To avoid noise caused by low presence OTUs, we removed those above zero values in the abundance data table (row_minocc filter = 3). Subsequently, the data were normalized to avoid effects produced by differences in sequencing depth. With this in mind, four methods were used for the construction of the networks. In order to obtain similarity measures, we used two dissimilarity indices (Bray Curtis and Kullback–Leibler) and two correlation indices (Pearson and Spearman). The first threshold was set to generate an initial network containing 50 positive edges and 50 negative edges, derived from the scores obtained in the four similarity measures. To generate the final network, we performed a permutation and bootstrapping with 100 iterations. The Cytoscape program was used to visualize the networks, as well as to obtain the number of nodes, clustering coefficient, average path length, and density. Finally, drivers from both networks were obtained using the Netshift program (Kuntal et al., 2019), available at https://web.rniapps.net/netshift/webpage.

## Results and Discussion

### Soil Elemental Composition in Different Cauquenes Tailing Zones

**Figure 1:**
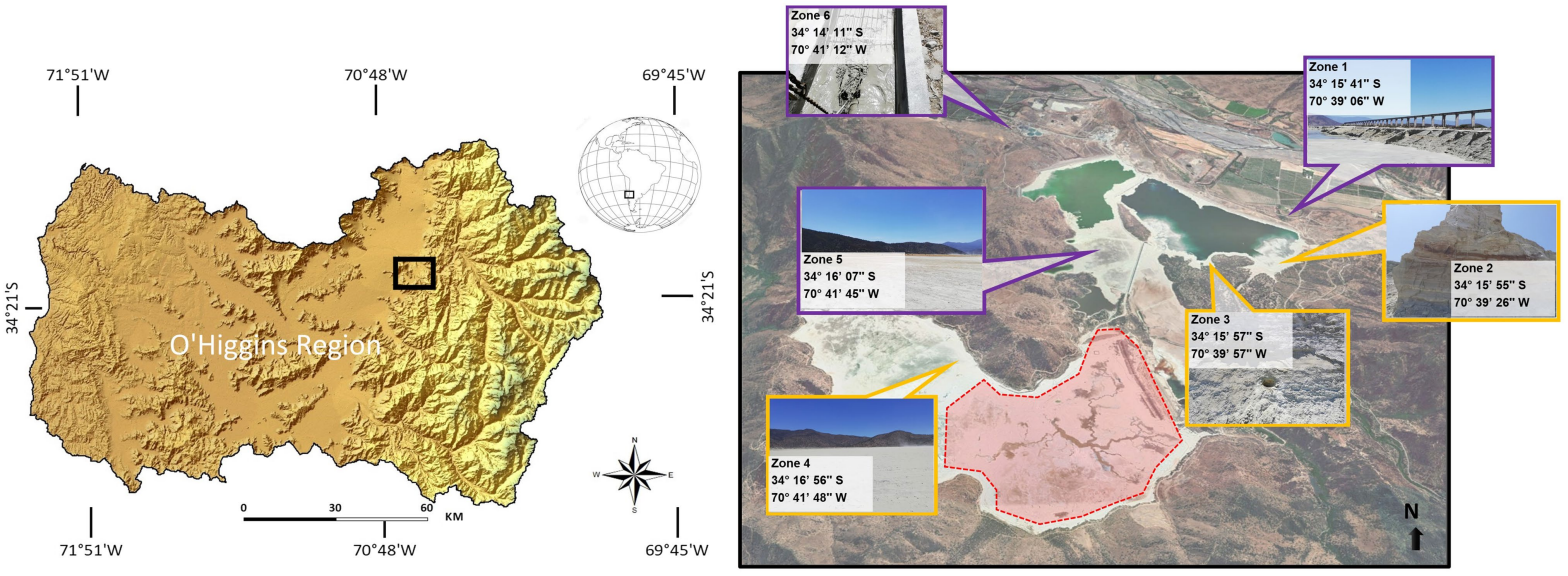
Geographical location of the study and sampling sites. Spatial coordinates of each sampling zone in the Cauquenes tailings. The red zone in the map corresponds to the miner operational labor (restricted access).

The Cauquenes tailings dam has an entire dimension of 12.5 km^2^ and is one of the oldest copper tailings in the world, containing 360 million tons of waste from copper mine exploitation, stored since 1936. To analyze the heavy metal concentration and pH, six different extraction points, from zone 1 (start site) to zone 6 (tail or end site), were selected, covering the principal location inside the tailing (Figure 1). Table 1 describes the different physicochemical characteristics of each sampled zone. We designated zones 1, 5, and 6 as “new tailings”; zones 2, 3, and 4 as “old tailings” and found great differences regarding the acidity of the soil and micronutrient concentrations. In particular, the old tailing zones had a higher concentration of Zn, Mn, and Fe compared to the other zones. Of special interest were the variations in acidity and Cu concentrations (highlighted in red), which, as previously stated, are environmental conditions in which acidophilic bacteria (such as bioleaching bacteria) can live and thrive (Kishimoto et al., 1991; Bond et al., 2000). Specifically, the old zones had an acidic soil pH and high Cu concentrations, while the new zones had relatively neutral soil pH and low Cu concentrations. Notably, zones 2 and 3 showed a higher concentration of Cu than other mining sites, such as those found in a heap leaching site from a copper mine in China (Xie et al., 2007), in water samples from a copper mine in China (He et al., 2007) and in copper mine waters from an abandoned copper mine in Wales (Hallberg et al., 2006). Mainly, zones 2 and 3 correspond to an old sector of the tailing, in which high concentrations of the mineral are expected, due to the extraction techniques employed at the beginning of the exploitation of “El Teniente” mine. On the contrary, zones 1, 5, and 6 correspond to the most recent exploitation (lasting no more than 30 years).

**Table 1:** Total elemental concentrations from soluble soil extracts.

Element (mg/L)	New tailing			Old tailing		
	Zone 1	Zone 5	Zone 6	Zone 2	Zone 3	Zone 4
Br	0 ± 0	0 ± 0	0 ± 0	0.8 ± 0	0.8 ± 0.1	0.1 ± 0
Ca	142.3 ± 17.2	64.9 ± 9.5	82 ± 2.5	665 ± 98.4	112.2 ± 9.8	69.1 ± 7.1
Cu	0 ± 0	0 ± 0	0 ± 0	1657.3 ± 211.9	315.6 ± 11.2	77.4 ± 12.8
Fe	0.1 ± 0	0.2 ± 0	0.5 ± 0.1	19.3 ± 1.1	58.7 ± 7.7	1.8 ± 0
K	22.3 ± 3.4	35.6 ± 2.8	19.1 ± 0.3	52 ± 2.6	26.6 ± 1.4	4.1 ± 0.6
Mn	0 ± 0	0 ± 0	0 ± 0	19 ± 0.8	200.9 ± 1.9	1.8 ± 0.1
Na	10.1 ± 0.2	12.5 ± 1.6	14.1 ± 0.6	207.8 ± 34.7	263.5 ± 19.1	32.3 ± 4.4
Ni	0 ± 0	0 ± 0	0 ± 0	12.8 ± 2.2	0.5 ± 0	0.2 ± 0
P	1.7 ± 0.1	0.9 ± 0.1	1.9 ± 0.2	68.9 ± 7	4.5 ± 0.4	1.7 ± 0.3
Zn	0 ± 0	0 ± 0	0.2 ± 0	184.8 ± 0	135.9 ± 0	0.9 ± 0
pH (unit)	6.3 ± 0.1	5.7 ± 0.1	6.6 ± 0.1	3.7 ± 0.1	3.3 ± 0.1	4.2 ± 0

Data correspond to the pH, and soluble micro- and macroelement concentrations present in the water extract from soil sampling zones.

**Figure 2:**
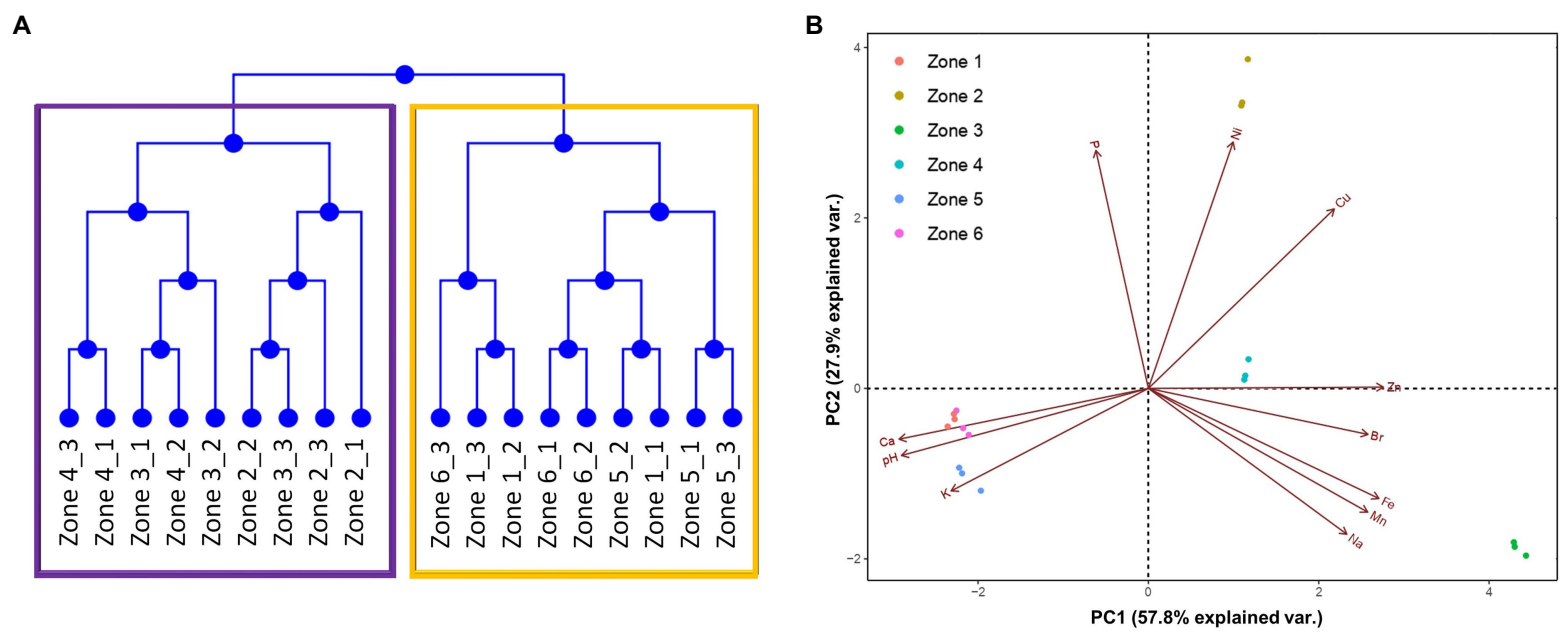
Similarity between each sampling site. **(A)** Dendrogram detailing the grouping of the six zones. **(B)** Principal component analysis of the physicochemical characteristics of each sampling site. Arrows represent how strongly each characteristic influences the principal components.

By performing clustering and principal component analysis (PCA), we found that the 6 zones can be grouped into two subgroups, according to the previously mentioned characteristics (Figures 2A,B). As expected, zones 1, 5, and 6 showed a very tight grouping, in both dendrograms and PCA, distancing themselves from the other zones. On the other hand, although zones 2, 3, and 4 were grouped together in the dendrogram, the PCA allowed us to spot some differences between them. For example, zones 2 and 4 were highly similar regarding PC1, but distanced themselves from zone 3. These three zones also showed different grouping in the PC2; as shown by the arrows in the figure, these differences could be due to the different concentrations of Ni, Fe, Mn, P, and Zn in these zones. This grouping may be due to the longevity of the sampled sites. As mentioned before, zones 2, 3, and 4 correspond to an old sector of the tailing, in which mineral concentrations are higher than in the other zones. On the other hand, the low concentrations of Cu measured in zones 1, 5, and 6 may reflect highly efficient extraction techniques that are currently employed, which allows little of the metal to remain in the waste.

### Microbiome Abundance and Diversity

**Figure 3:**
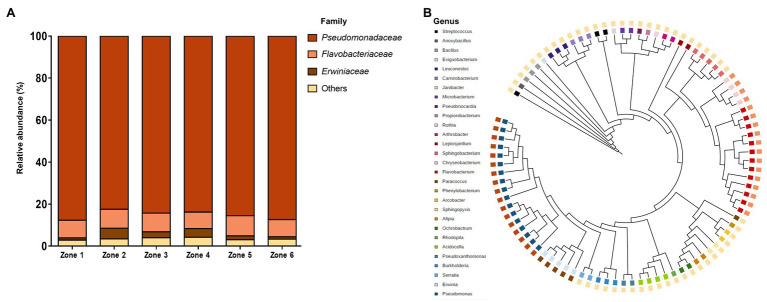
Taxonomic analysis of the bacterial community. **(A)** Relative abundance of soil bacterial families based on massive sequencing and OTU assignments in the six sampled zones. Others: relative abundance <5%. **(B)** Phylogenetic tree of bacterial networks from the six sampled zones. Tree colors illustrate the family of each OTU.

The taxonomic classification of the bacterial community in the sampled zones encompassed 17 families (Figure 3A). The relative abundance in all zones was rather low, represented mainly by three bacterial families: *Pseudomonadaceae*, *Flavobacteriaceae*, and *Erwiniaceae*. We expected to find a higher diversity in the neutral-pH zones (zones 1, 5, and 6), since other studies had previously reported that neutral-pH environments exhibit greater bacterial diversity when compared to with acidic soil environments, such as those of zones 2, 3, and 4 (Fierer and Jackson, 2006; Xiao et al., 2021). In terms of representation, there was an overrepresentation of the *Pseudomonadaceae* family in the six zones, an expected result considering previous results from different heavy metal soil samples, including mine tailings (Gagnon et al., 2020; Huang et al., 2021). The relative abundance of the *Erwiniaceae* family differed between zones, having a higher abundance in zones 2, 3, and 4 (old tailings). The high concentration of metals (mainly Cu) could explain this shift in abundance. There are only a few reports about *Erwiniaceae* family survival on high Cu concentrations (Sholberg et al., 2001; Vegetale and Pathology, 2016), which also support their higher representation in the older zones. The relative abundance of the *Flavobacteriaceae* family remained mainly constant in the six sampled zones, and the “other” groups were composed of *Micrococcaceae*, *Oxalobacteraceae*, and *Acetobacteraceae*, among others. Interestingly, we were able to identify bacterial species with biotechnological potentials, such as bioleaching of metals (*Erwinia* spp.; Tsuruta, 2004), alleviation of drought stress in crop growth (*Arthrobacter spp.*; Nordstedt and Jones, 2020), biodegradation of oil (*Pseudomonas spp.*; Abalos et al., 2004), and biosynthesis of secondary metabolites (*Flavobacteriia spp.*; Kraut-cohen et al., 2021), among others. Overall, different soil samples of the same tailing can harbor different types of microbial communities, and these differences may be due to the acidity of the environment or micronutrient concentrations. This result was expected, since variations in soil pH are related to changes in bacterial communities (Lauber et al., 2009; Mandakovic et al., 2018). In addition, our results show that the relative abundance of bacterial families in this extreme environment is rather low, confirming the results of microbial diversity in acid mine drainages (Baker and Banfield, 2003; Tyson et al., 2004), or in heavy metal-contaminated soils (Li et al., 2017; Tseng et al., 2021). Additionally, the soil micronutrient composition or other environmental factors, such as temperature or oxygen availability, could explain the low relative abundance of some bacteria (Landesman et al., 2014; Freedman and Zack, 2015).

When the genera were analyzed in the new and old tailing, the results were like those observed previously, where the predominant genera are Pseudomonas, Flavobacterium, and Erwinia, occupying more than 95% of the total abundance, with patterns like those mentioned above. Other members of the community, which have an abundance of less than 1% but have a higher abundance in the old tailing’s areas, belong to the genera Acidocella, Burkholderia, and Serratia. In this regard, it has been observed that members of the genus Acidocella are commonly found in acid tailings and can reduce different metals (Sağlam et al., 2016). Similarly, it has been observed that Burkholderia and Serratia have the ability to resist high concentrations of heavy metals, such as cadmium for Burkholderia and cadmium, cobalt, and zinc for Serratia (Zelaya-Molina et al., 2016; Wang et al., 2019).

These results indicate the great biotechnological potential in the members of the bacterial community, in genera of great abundance, as well as in different members of the community with a small representation in the bacterial environment.

Phylogenetic and taxonomical relationships between each OTU were visualized with the iTOL tool (Figure 3B). OTUs belonging to the “Other” families were the most predominant, *Pseudomonadaceae* and *Flavobacteriaceae* were the second most prevalent, and *Erwinia* was the least predominant one. Relative bacterial abundance and bacterial diversity from the sampled sites were drastically different. On the one hand, the relative abundance was dominated by bacteria from the *Pseudomonadaceae* family, while bacterial diversity was led by bacteria from the “Other” families. This may be attributed to the number of bacterial species belonging to each family (i.e., the *Pseudomonadaceae* family had fewer bacterial species than the “Other” families). We also calculated the Shannon index and evenness to evaluate the diversity of each zone. Both values were similar for each sample (~2 and ~ 0.35, respectively). These values, together with the abundance values, indicate that although the samples harbor a diverse microbiome, the highest percentage of abundance was occupied by a few OTUs, which in this case, belong to the *Pseudomonadaceae* family.

### Co-occurrence Network Models of the New and Old Tailings

**Figure 4:**
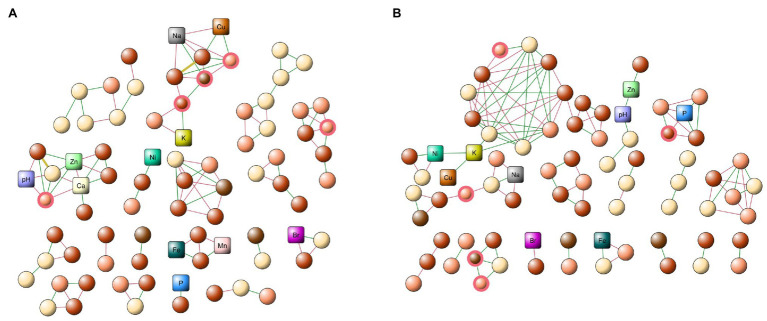
Microbial co-occurrence networks. **(A)** Old tailing network, composed of zones 2, 3, and 4. **(B)** New tailing network, composed of zones 1, 5, and 6. Both networks were constructed using the abundance of each site, as well as the micronutrients and pH, respectively. The square nodes correspond to the physicochemical values, and the circular nodes, to the respective OTUs. For the latter, color nomenclature was defined based on the family of each out: light Brown, *Pseudomonadaceae*; dark Brown *Erwiniaceae;* light salmon *Flavobacteriaceae;* and wheat Other families. Green edge indicates a positive correlation and the red edge, a negative one. The red node border highlights the driver member.

To determine the interactions of the microbiome communities, we constructed co-occurrence networks. Figure 4 shows the models constructed using the abundances of each OTU, also including the physicochemical properties of each zone (pH and element concentrations). Both networks presented a similar structure in the number of nodes (80 and 76 for networks 1 and 2, respectively), as well as a similar number of edges (106 and 108, respectively). When comparing the networks, we observed that both of them shared most of the nodes (more than 80%). However, only 2% of the edges (connectivity) were similar in both networks, indicating the occurrence of significant reconfiguration in the interactions established by the members of the microbiome between the networks.

When analyzing the topological properties, we observed that they present a large number of common elements in both networks, such as a characteristic path length (1.893 and 1.934, respectively), density (0.429 and 0.451, respectively), heterogeneity (0.333 and 0.358, respectively), and centralization (0.190 and 0.192, respectively). Interestingly, we observed a change in the value of the clustering coefficient (0.521 and 0.789, respectively), indicating that although both networks present a significant amount of similarities, a reordering mechanism changes the interactions between the members of the networks.

In general, we observed an evident fragmentation inside the community in the old tailings network, which could be related to the change of soil acidity and the high concentration of metals present. In this regard, a study conducted in Mountain Gongga, China (Li et al., 2020) showed similar results, where sites with a higher soil pH had a more connected network, while a decreased pH caused a reduction in network collapse, thus decreasing network interactions. In addition, soil pH could shift a series of intracellular biochemical pathways, impacting microbial enzymatic activities (Stark et al., 2014), which could then affect the indirect interactions established between the members of the microbiome, through either the decrease of secondary metabolites or harmful molecules for the members of the interaction network.

In terms of the interactions between the OTUs and the abiotic characteristics, we observed Cu, Zn, Na, and Ca the elements and a variable pH in the old tailings, which are highly connected with members of the community. In particular, Cu and Zn present a greater positive interaction with OTUs from the *Flavobacteria* and *Gammaproteobacteria* class, as well as a greater positive interaction between both classes. As shown in Table 1, these elements in the older tailings are present at high concentrations. An increase in these positive interactions may be due to the increase in the concentration of metals in the old tailings, suggesting commensalism between species, which allows greater adaptability to environments with high concentrations of metals, especially Cu. Accordingly, previous studies in a Cu mine drainage in the Jinsha River, China showed that there is an increase in positive interactions between members of the microbiome contaminated with large amounts of Cu and other metals, compared to uncontaminated areas (Yuan et al., 2021). Therefore, an excess of metals can produce a change in microbial interactions, increasing positive interactions between members of the microbiome (Yin et al., 2015; Li et al., 2017). Furthermore, these interactions allow better adaptation and growth of bacteria in environments with large amounts of metals (Pande and Kost, 2017).

To determine putative keystone nodes in the networks, we used the Netshift application (Kuntal et al., 2019). This application allowed us to identify only drivers between the networks (previously defined as conserved nodes in both networks), which significantly change their connectivity with other members of the community when comparing the old and new tailings. We found five key nodes, of which three belonged to the *Flavobacteria* genus, one corresponded to the *Erwinia* genus and the last one belonged to the *Pseudomonas*. These key nodes also showed an increase in positive interactions in the network of the old tailings, compared to the new tailings, indicating a possible increase in mutualistic relationships between the key nodes and their direct neighborhood (Faust and Raes, 2012). As previously reported, members of the *Flavobacteria* genus are capable of producing different types of secondary metabolites, which can participate in different cell protection pathways, such as oxidative stress (Nishimura et al., 2017; Enisoglu-Atalay et al., 2018), which is normally increased in the extreme environment of the old tailings, in order to help the bacterial community to increase its resistance to hostile environments. As reported, members from the *Erwinia* genus exhibit properties that allow them to survive in these hostile environments; these bacteria also participate as key nodes in the network, including the ability to bioleach and reduce different metals (Tsuruta, 2004). In the case of the *Pseudomonas* genus, they present a high resistance to metals and participate in their reduction (Huang et al., 2016), allowing them to generate a better microenvironment for their bacterial neighborhood. Of note, this characteristic highlights their possible use in biotechnology for soil remediation. The *Flavobacteria* and *Erwinia* genera only occupied between 1 and 8% of the observed abundance, indicating the importance of the rare taxa within the bacterial community. Finally, we were able to identify a total of 32 OTUs belonging to rare taxes, so future studies are necessary to further understand the participation of this bacterial species in the miner communities (Campbell et al., 2011; Jousset et al., 2017).

## Conclusion

In this study, samples from six different sectors of the “Cauquenes” copper mine tailings were evaluated in order to obtain a better understanding of the extreme environment in which extremophile bacteria survive. By doing several *in silico* analyses, we found that the tailings could be divided in two different sectors: the new tailings, which has a neutral pH and low metal concentration, and the old tailings, which has high metal concentration and acidic pH. In general, the bacterial abundance was low in both sectors, represented mainly by the family *Pseudomonadaceae*, followed by the *Flavobacteriaceae* and *Erwiniaceae* families. Bacterial diversity was led by bacterial species from the “Other” bacterial family group (which encompassed families with <%5 of relative abundance). By constructing co-occurrence networks, we determined the interactions of the microbial community and revealed the occurrence of rearrangements in the interactions between the new and the old tailings, which is related to the change in metal concentrations and the decrease in pH of the old tailings We also detected five keystone nodes in the networks, which also promise an interesting biotechnological potential. In summary, our work provided one of the first studies of microbiome description in mine tailings, providing novel insights about the interactions of the microbial community in extreme environments.

## Data Availability Statement

All sequence data used in this study have been deposited in the Sequence Read Archive (SRA) of the National Center for Biotechnology Information (NCBI) under the BioProject accession number PRJNA769703 (https://www.ncbi.nlm.nih.gov/sra/?term=PRJNA769703).

## Author Contributions

GG, JO, and ML performed all the bioinformatics, analyzed the data, and wrote the manuscript. FF made the soil chemical assays. VA-T, AR-J, VP, and LP contributed to the DNA extractions, *in silico* protocols, and discussion. ML has full responsibility for the article. All authors contributed to the article and approved the submitted version.

## Funding

This study was supported by the Center for Mathematical Modeling, Apoyo a Centros de Excelencia ACE210010; Proyecto ANILLO regular ANID ACT210004; Fondo Basal FB210005; FONDAP grants 15090007 and 15130011; FONDECYT grants 11200934, 1190742, and 1190743, Proyecto Gobierno Regional O’Higgins FIC IDI40008909-0; and Fondo Interdisciplinario Interno UOH.

## Conflict of Interest

The authors declare that the research was conducted in the absence of any commercial or financial relationships that could be construed as a potential conflict of interest.

## Publisher’s Note

All claims expressed in this article are solely those of the authors and do not necessarily represent those of their affiliated organizations, or those of the publisher, the editors and the reviewers. Any product that may be evaluated in this article, or claim that may be made by its manufacturer, is not guaranteed or endorsed by the publisher.

## References

[ref1] Abalos A. Viñas M. Sabaté J. Manresa M. A. Solanas A. M. (2004). Enhanced biodegradation of Casablanca crude oil by a microbial consortium in presence of a rhamnolipid produced by Pseudomonas aeruginosa AT10. Biodegradation 15, 249–260. doi: 10.1023/b:biod.0000042915.28757.fb, PMID: 15473554

[ref2] Akinci G. Guven D. E. (2011). Bioleaching of heavy metals contaminated sediment by pure and mixed cultures of Acidithiobacillus spp. Desalination 268, 221–226. doi: 10.1016/j.desal.2010.10.032

[ref4] Alvarez S. Jerez C. A. (2004). Copper ions stimulate polyphosphate degradation and phosphate efflux in Acidithiobacillus ferrooxidans. Appl. Environ. Microbiol. 70, 5177–5182. doi: 10.1128/AEM.70.9.5177-5182.2004, PMID: 15345397 PMC520870

[ref5] Antranikian G. Vorgias C. E. Bertoldo C. (2005). Extreme environments as a resource for microorganisms and novel biocatalysts. Adv. Biochem. Eng. Biotechnol. 96, 219–262. doi: 10.1007/b135786, PMID: 16566093

[ref6] Baker B. J. Banfield J. F. (2003). Microbial communities in acid mine drainage. FEMS Microbiol. Ecol. 44, 139–152. doi: 10.1016/S0168-6496(03)00028-X 19719632

[ref7] Bond P. L. Druschel G. K. Banfield J. F. (2000). Comparison of acid mine drainage microbial communities in physically and geochemically distinct ecosystems. Appl. Environ. Microbiol. 66, 4962–4971. doi: 10.1128/AEM.66.11.4962-4971.2000, PMID: 11055950 PMC92406

[ref8] Bordron P. Latorre M. Cortés M. P. González M. Thiele S. Siegel A.. (2016). Putative bacterial interactions from metagenomic knowledge with an integrative systems ecology approach. Microbiology 5, 106–117. doi: 10.1002/mbo3.315, PMID: 26677108 PMC4767419

[ref9] Campbell B. J. Yu L. Heidelberg J. F. Kirchman D. L. (2011). Activity of abundant and rare bacteria in a coastal ocean. Proc. Natl. Acad. Sci. 108, 12776–12781. doi: 10.1073/pnas.1101405108, PMID: 21768380 PMC3150899

[ref10] Caporaso J. G. Kuczynski J. Stombaugh J. Bittinger K. Bushman F. D. Costello E. K.. (2011). QIIME allows analysis of high-throughput community sequencing data. Nat. Methods 7, 335–336. doi: 10.1038/nmeth.f.303.QIIME PMC3156573 20383131

[ref11] Chung A. P. Coimbra C. Farias P. Francisco R. Branco R. Simão F. v., . (2019). Tailings microbial community profile and prediction of its functionality in basins of tungsten mine. Sci. Rep. 9. doi: 10.1038/s41598-019-55706-6, 19596, PMID: 31862994 PMC6925229

[ref12] Edgar R. C. (2010). Search and clustering orders of magnitude faster than BLAST. Bioinformatics 26, 2460–2461. doi: 10.1093/bioinformatics/btq461, PMID: 20709691

[ref13] Edwards K. J. Goebel B. M. Rodgers T. M. Schrenk M. O. Gihring T. M. Cardona M. M.. (1999). Geomicrobiology of pyrite (Fes2) dissolution: case study at iron mountain. J. Geomicrobiol. 16, 155–179. doi: 10.1080/014904599270668

[ref14] Enisoglu-Atalay V. Atasever-arslan B. Yaman B. Cebecioglu R. Kul A. Ozilhan S.. (2018). Chemical and molecular characterization of metabolites from Flavobacterium sp. PLoS One 13:e0205817. doi: 10.1371/journal.pone.0205817, PMID: 30332474 PMC6192653

[ref15] Faust K. Raes J. (2012). Microbial interactions: from networks to models. Nat. Rev. Microbiol. 10, 538–550. doi: 10.1038/nrmicro2832 22796884

[ref16] Faust K. Raes J. (2016). CoNet app: inference of biological association networks using Cytoscape [version 1; referees: 2 approved with reservations]. F1000Res. 5:1519. doi: 10.12688/f1000research.9050.1 27853510 PMC5089131

[ref17] Fierer N. Jackson R. B. (2006). The diversity and biogeography of soil bacterial communities. Proc. Natl. Acad. Sci. 103, 626–631. doi: 10.1073/pnas.0507535103, PMID: 16407148 PMC1334650

[ref18] Freedman Z. Zack D. R. (2015). Soil bacterial communities are shaped by temporal and environmental filtering: evidence from a long-term chronosequence. Environ. Microbiol. 17, 3208–3218. doi: 10.1111/1462-2920.12762, PMID: 25581568

[ref19] Gagnon V. Rodrigue-Morin M. Tremblay J. Wasserscheid J. Champagne J. Bellenger J. P.. (2020). Life in mine tailings: microbial population structure across the bulk soil, rhizosphere, and roots of boreal species colonizing mine tailings in northwestern Québec. Ann. Microbiol. 70, 1–18. doi: 10.1186/s13213-020-01582-9

[ref20] González-Toril E. Águilera Á. Souza-Egipsy V. Pamo E. L. España J. S. Amils R. (2011). Geomicrobiology of La Zarza-Perrunal acid mine effluent (Iberian Pyritic Belt, Spain). Appl. Environ. Microbiol. 77, 2685–2694. doi: 10.1128/AEM.02459-10, PMID: 21357431 PMC3126378

[ref21] Hallberg K. B. Coupland K. Kimura S. Johnson D. B. (2006). Macroscopic streamer growths in acidic, metal-rich mine waters in North Wales consist of novel and remarkably simple bacterial communities. Appl. Environ. Microbiol. 72, 2022–2030. doi: 10.1128/AEM.72.3.2022-2030.2006, PMID: 16517651 PMC1393227

[ref22] Handl S. Dowd S. E. Garcia-mazcorro J. F. Steiner J. M. Suchodolski J. S. (2011). Massive parallel 16S rRNA gene pyrosequencing reveals highly diverse fecal bacterial and fungal communities in healthy dogs and cats. FEMS Microbiol. Ecol. 76, 301–310. doi: 10.1111/j.1574-6941.2011.01058.x, PMID: 21261668

[ref23] Hart A. Cortés M. P. Latorre M. Martinez S. (2018). Codon usage bias reveals genomic adaptations to environmental conditions in an acidophilic consortium. PLoS One 13:e0195869. doi: 10.1371/journal.pone.0195869, PMID: 29742107 PMC5942774

[ref24] He Z. Xie X. Xiao S. Liu J. Qiu G. (2007). Microbial diversity of mine water at Zhong Tiaoshan copper mine. China. J. Basic Microbiol. 47, 485–495. doi: 10.1002/jobm.200700219, PMID: 18072249

[ref25] Huang C. C. Liang C. M. Yang T. I. Chen J. L. Wang W. K. (2021). Shift of bacterial communities in heavy metal-contaminated agricultural land during a remediation process. PLoS One 16:e0255137. doi: 10.1371/journal.pone.0255137, PMID: 34297781 PMC8301633

[ref26] Huang H. Wu K. Khan A. Jiang Y. Ling Z. Liu P.. (2016). A novel pseudomonas gessardii strain LZ-E simultaneously degrades naphthalene and reduces hexavalent chromium. Bioresour. Technol. 207, 370–378. doi: 10.1016/j.biortech.2016.02.015, PMID: 26901089

[ref27] Jousset A. Bienhold C. Chatzinotas A. Gallien L. Gobet A. Kurm V.. (2017). Where less may be more: how the rare biosphere pulls ecosystems strings. ISME J. 11, 853–862. doi: 10.1038/ismej.2016.174, PMID: 28072420 PMC5364357

[ref3] Kishimoto N. Kosako Y. Tano T. (1991). Acidobacterium capsulatum gen. nov., sp. nov.: An acidophilic chemoorganotrophic bacterium containing menaquinone from acidic mineral environment. Curr. Microbiol. 22, 1–7. doi: 10.1007/BF02106205 23835745

[ref28] Kraut-cohen J. Shapiro O. H. Dror B. Cytryn E. (2021). Pectin induced Colony expansion of soil-derived Flavobacterium strains. Front. Microbiol. 12:544. doi: 10.3389/fmicb.2021.651891, PMID: 33889143 PMC8056085

[ref29] Kuntal B. K. Chandrakar P. Sadhu S. Mande S. S. (2019). ‘NetShift’: a methodology for understanding ‘driver microbes’ from healthy and disease microbiome datasets. ISME J. 13, 442–454. doi: 10.1038/s41396-018-0291-x, PMID: 30287886 PMC6331612

[ref30] Landesman W. J. Nelson D. M. Fitzpatrick M. C. (2014). Soil properties and tree species drive ß -diversity of soil bacterial communities. Soil Biol. Biochem. 76, 201–209. doi: 10.1016/j.soilbio.2014.05.025

[ref31] Latorre M. Cortés M. P. Travisany D. Di Genova A. Budinich M. Reyes-Jara A.. (2016). The bioleaching potential of a bacterial consortium. Bioresour. Technol. 218, 659–666. doi: 10.1016/j.biortech.2016.07.012, PMID: 27416516

[ref32] Lauber C. L. Hamady M. Knight R. Fierer N. (2009). Pyrosequencing-based assessment of soil pH as a predictor of soil bacterial community structure at the continental scale. Appl. Environ. Microbiol. 75, 5111–5120. doi: 10.1128/AEM.00335-09, PMID: 19502440 PMC2725504

[ref33] Letunic I. Bork P. (2021). Interactive tree Of life (iTOL) v5: an online tool for phylogenetic tree display and annotation. Nucleic Acids Res. 49, W293–W296. doi: 10.1093/nar/gkab301, PMID: 33885785 PMC8265157

[ref34] Li J. Li C. Kou Y. Yao M. Jie H. Z. Li X. (2020). Distinct mechanisms shape soil bacterial and fungal co-occurrence networks in a mountain ecosystem. FEMS Microbiol. Ecol. 96:fiaa030. doi: 10.1093/femsec/fiaa030, PMID: 32109277

[ref35] Li X. Meng D. Li J. Yin H. Liu H. Liu X.. (2017). Response of soil microbial communities and microbial interactions to long-term heavy metal contamination. Environ. Pollut. 231, 908–917. doi: 10.1016/j.envpol.2017.08.057, PMID: 28886536

[ref36] Mandakovic D. Rojas C. Maldonado J. Latorre M. Travisany D. Delage E.. (2018). Structure and co-occurrence patterns in microbial communities under acute environmental stress reveal ecological factors fostering resilience. Sci. Rep. 8, 5812–5875. doi: 10.1038/s41598-018-23931-0, PMID: 29651160 PMC5897386

[ref37] Mcdonald D. Price M. N. Goodrich J. Nawrocki E. P. Desantis T. Z. Probst A.. (2011). An improved Greengenes taxonomy with explicit ranks for ecological and evolutionary analyses of bacteria and archaea. ISME J. 6, 610–618. doi: 10.1038/ismej.2011.139, PMID: 22134646 PMC3280142

[ref38] Moore C. M. Helmann J. D. (2005). Metal ion homeostasis in Bacillus subtilis. Curr. Opin. Microbiol. 8, 188–195. doi: 10.1016/j.mib.2005.02.007 15802251

[ref39] Navarro C. A. Von Bernath D. Jerez C. (2013). Heavy metal resistance strategies of acidophilic bacteria and their acquisition: importance for biomining and bioremediation. Biol. Res. 46, 363–371. doi: 10.4067/S0716-97602013000400008, PMID: 24510139

[ref40] Nishimura K. Matsumoto R. Yonezawa Y. Nakagawa H. (2017). Effect of quercetin on cell protection via erythropoietin and cell injury of HepG2 cells. Arch. Biochem. Biophys. 636, 11–16. doi: 10.1016/j.abb.2017.10.013, PMID: 29080630

[ref41] Nordstedt N. P. Jones M. L. (2020). Isolation of Rhizosphere bacteria That improve quality and water stress tolerance in greenhouse ornamentals. Front. Plant Sci. 11:826. doi: 10.3389/fpls.2020.00826, PMID: 32612623 PMC7308537

[ref42] Oluwasola E. A. Hainin M. R. Aziz M. M. A. Yaacob H. Warid M. N. M. (2014). Potentials of steel slag and copper mine tailings as construction materials. Mater. Res. Innov. 18, 250–254. doi: 10.1179/1432891714Z.000000000966

[ref43] Orellana R. Macaya C. Bravo G. Dorochesi F. Cumsille A. Valencia R.. (2018). Living at the Frontiers of life: extremophiles in Chile and their potential for bioremediation. Front. Microbiol. 9:2309. doi: 10.3389/fmicb.2018.02309, PMID: 30425685 PMC6218600

[ref44] Pande S. Kost C. (2017). Bacterial Unculturability and the formation of intercellular metabolic networks. Trends Microbiol. 25, 349–361. doi: 10.1016/j.tim.2017.02.015, PMID: 28389039

[ref45] Porcheron G. Garénaux A. Proulx J. Sabri M. Dozois M. C. (2013). Iron, copper, zinc, and manganese transport and regulation in pathogenic Enterobacteria: correlations between strains, site of infection and the relative importance of the different metal transport systems for virulence. Front. Cell. Infect. Microbiol. 3:90. doi: 10.3389/fcimb.2013.00090, PMID: 24367764 PMC3852070

[ref46] Rawlings D. E. (2005). Characteristics and adaptability of iron- and sulfur-oxidizing microorganisms used for the recovery of metals from minerals and their concentrates. Microb. Cell Factories 4, 1–15. doi: 10.1186/1475-2859-4-13, PMID: 15877814 PMC1142338

[ref47] Russell J. B. Cook G. M. (1995). Energetics of bacterial growth: balance of anabolic and catabolic reactions. Microbiol. Rev. 59, 48–62. doi: 10.1128/mr.59.1.48-62.1995, PMID: 7708012 PMC239354

[ref48] Sağlam E. S. Akçay M. Çolak D. N. İnan Bektaş K. Beldüz A. O. (2016). Generation of acid mine drainage around the Karaerik copper mine (Espiye, Giresun, NE Turkey): implications from the bacterial population in the Acısu effluent. Extremophiles 20, 673–685. doi: 10.1007/s00792-016-0857-3, PMID: 27338270

[ref49] Santofimia E. González-Toril E. López-Pamo E. Gomariz M. Amils R. Aguilera Á. (2013). Microbial diversity and its relationship to physicochemical characteristics of the water in two extreme acidic pit lakes from the Iberian Pyrite Belt (SW Spain). PLoS One 8:e66746. doi: 10.1371/journal.pone.0066746, PMID: 23840525 PMC3694112

[ref50] Shannon P. Markiel A. Ozier O. Baliga N. S. Wang J. T. Ramage D.. (2003). Cytoscape: A software environment for integrated models of biomolecular interaction networks. Genome Res. 13, 2498–2504. doi: 10.1101/gr.1239303, PMID: 14597658 PMC403769

[ref51] Sholberg P. L. Bedford K. E. Haag P. Randall P. (2001). Survey of Erwinia amylovora isolates from British Columbia for resistance to bactericides and virulence on apple. Can. J. Plant Pathol. 23, 60–67. doi: 10.1080/07060660109506910

[ref52] Stark S. Männistö M. K. Eskelinen A. (2014). Nutrient availability and pH jointly constrain microbial extracellular enzyme activities in nutrient-poor tundra soils. Plant Soil 383, 373–385. doi: 10.1007/s11104-014-2181-y

[ref53] Tapia L. Suazo M. Hödar C. Cambiazo V. González M. (2003). Copper exposure modifies the content and distribution of trace metals in mammalian cultured cells. Biometals 16, 169–174. doi: 10.1023/a:1020766932605, PMID: 12572676

[ref54] Teng W. Kuang J. Luo Z. Shu W. (2017). Microbial diversity and community assembly across environmental gradients in acid mine drainage. Fortschr. Mineral. 7:106. doi: 10.3390/min7060106

[ref55] Thomas L. V. Wimpenny J. W. T. (1996). Investigation of the effect of combined variations in temperature, pH, and NaCl concentration on Nisin inhibition of listeria monocytogenes and Staphylococcus aureus. Appl. Environ. Microbiol. 62, 2006–2012. doi: 10.1111/j.1550-7408.1999.tb04612.x, PMID: 8787399 PMC167979

[ref56] Tseng S. Liang C. Chia T. Ton S. (2021). Changes in the composition of the soil bacterial community in heavy metal-contaminated farmland. Int. J. Environ. Res. Public Health 18:8661. doi: 10.3390/ijerph18168661, PMID: 34444410 PMC8394363

[ref57] Tsuruta T. (2004). Biosorption and recycling of gold using various microorganisms. J. Gen. Appl. Microbiol. 50, 221–228. doi: 10.2323/jgam.50.221, PMID: 15754248

[ref58] Turner S. Pryerb K. M. Miao V. P. W. Palmera J. D. (1999). Investigating deep phylogenetic relationships among cyanobacteria and plastids by small subunit rRNA sequence analysis. J. Eukaryot. Microbiol. 46, 327–338. doi: 10.1111/j.1550-7408.1999.tb04612.x, PMID: 10461381

[ref59] Tyson G. W. Chapman J. Hugenholtz P. Allen E. E. Ram R. J. Richardson P. M.. (2004). Community structure and metabolism through reconstruction of microbial genomes from the environment. Nature 428, 37–43. doi: 10.1038/nature02340, PMID: 14961025

[ref60] Valdés J. Pedroso I. Quatrini R. Dodson R. J. Tettelin H. Blake R.. (2008). Acidithiobacillus ferrooxidans metabolism: From genome sequence to industrial applications. BMC Genomics 9, 1–24. doi: 10.1186/1471-2164-9-597, PMID: 19077236 PMC2621215

[ref61] Wang X. Zhang X. Liu X. Huang Z. Niu S. Xu T.. (2019). Physiological, biochemical and proteomic insight into integrated strategies of an endophytic bacterium: Burkholderia cenocepacia strain YG-3 response to cadmium stress. Metallomics 11, 1252–1264. doi: 10.1039/c9mt00054b, PMID: 31173023

[ref62] Xiao E. Ning Z. Xiao T. Sun W. Jiang S. (2021). Soil bacterial community functions and distribution after mining disturbance. Soil Biol. Biochem. 157:108232. doi: 10.1016/j.soilbio.2021.108232

[ref63] Xie X. Xiao S. He Z. Liu J. Qiu G. (2007). Microbial populations in acid mineral bioleaching systems of Tong Shankou copper mine. China. J. Appl. Microbiol. 103, 1227–1238. doi: 10.1111/j.1365-2672.2007.03382.x, PMID: 17897227

[ref64] Yang T. Liu J. Chen W. C. E. Chen X. Shu Y. H. Jia P.. (2017). Changes in microbial community composition following phytostabilization of an extremely acidic cu mine tailings. Soil Biol. Biochem. 114, 52–58. doi: 10.1016/j.soilbio.2017.07.004

[ref65] Yin H. Niu J. Ren Y. Cong J. Zhang X. (2015). An integrated insight into the response of sedimentary microbial communities to heavy metal contamination. Sci. Rep. 5, 1–12. doi: 10.1038/srep14266, PMID: 26391875 PMC4585741

[ref66] Yuan Q. Wang P. Wang C. Chen J. Wang X. Liu S. (2021). Indicator species and co-occurrence pattern of sediment bacterial community in relation to alkaline copper mine drainage contamination. Ecol. Indic. 120:106884. doi: 10.1016/j.ecolind.2020.106884

[ref67] Zelaya-Molina L. X. Hernández-Soto L. M. Guerra-Camacho J. E. Monterrubio-López R. Patiño-Siciliano A. Villa-Tanaca L.. (2016). Ammonia-oligotrophic and Diazotrophic heavy metal-resistant Serratia liquefaciens Strains from Pioneer plants and mine tailings. Microb. Ecol. 72, 324–346. doi: 10.1007/s00248-016-0771-3, PMID: 27138047

[ref68] Zhang Q. Banda J. F. Dong H. Hao C. Guo D. Mao W.. (2021). Responses of acidophilic communities in different acid mine drainages to environmental conditions in Nanshan mine, Anhui Province, China. Geomicrobiol J. 38, 686–697. doi: 10.1080/01490451.2021.1937405

[ref69] Zhou J. Bruns M. A. Tiedje J. M. (1996). DNA recovery from soils of diverse composition. Appl. Environ. Microbiol. 62, 316–322. doi: 10.1128/aem.62.2.316-322.1996, PMID: 8593035 PMC167800

